# The value of dynamic MRI in the treatment of cervical spondylotic myelopathy: a protocol for a prospective randomized clinical trial

**DOI:** 10.1186/s12891-020-3106-y

**Published:** 2020-02-07

**Authors:** Nanfang Xu, Youyu Zhang, Guangjin Zhou, Qiang Zhao, Shaobo Wang

**Affiliations:** 1 Department of Orthopaedics, Peking University Third Hospital, Beijing, People’s Republic of China; 2 Department of Radiology, Peking University Third Hospital, Beijing, People’s Republic of China

**Keywords:** Dynamic magnetic resonance image (dMRI), Cervical spondylotic myelopathy (CSM), Surgical treatment

## Abstract

**Background:**

Cervical spondylotic myelopathy (CSM) is the most severe type of cervical spondylosis and the most common cause of spinal cord dysfunction among adults over 55 years old. MRI plays an important role in the diagnosis and evaluation of CSM, which can directly demonstrate the correlation between disc, spinal cord, posterior structures and abnormal signal in spinal cord. Static MRI can only show the static and neutral position of spinal cord, which is not enough to understand the pathogenesis of CSM. Dynamic MRI demonstrating the extension and flexion position of spinal cord can be a better tool for the treatment of CSM, especially the surgical decision making.

**Method:**

A total of 180 CSM patients who have indications for surgery will be recruited in outpatient of Peking University Third Hospital and assigned to three groups (Group A, B and C) based on their static MRI after consent. Group A (incomplete dura compression) means the signal of cerebral spinal fluid (CSF) is still visible. Group B (complete dura compression) means no CSF signal and no shape change of spinal cord. Group C (spinal cord compression) means shape change of spinal cord. Two surgical plans will be made for each participant by one professional surgeon according to the static MRI and dynamic MRI respectively and we will randomly choose one to perform via a random number system. Follow-up will be maintained at 3, 6, and 12 months after surgery through outpatient or telephone interview, including mJOA score, 10-s G&R (grip and release) and 10-s step test, SF-36 score, radiographic examination and complications. Finally, data collection and statistical analysis will be finished by researchers who are blinded to recruitment and treatment.

**Discussion:**

This study will help us to explore the indication of dynamic MRI and the value of dynamic MRI in the treatment of CSM, especially the surgical decision making. Dynamic MRI can be a useful tool in the treatment of CSM patients.

**Trial registration:**

ChiCTR1900023014. Registered on May 7th, 2019.

## Background

Cervical spondylotic myelopathy (CSM) is the most severe type of cervical spondylosis and the most common cause of spinal cord dysfunction among adults over 55 years old [1–4]. The clinical syndrome of CSM occurs when the stenosis impinges on the spinal cord, and the severity of CSM is generally thought to be related to the amount of mechanical compression of the various spinal cord tracts [5]. The symptom of CSM ranges from mild impact on the daily life to paralysis. Decompression surgery is the most appropriate treatment after the diagnosis of CSM, which includes anterior, posterior and anterior-posterior approaches [6, 7]. The diagnosis of CSM is based on clinical symptoms, physical and radiological examination including x-ray, computed-tomography (CT) and magnetic resonance image (MRI) [5]. Compare to x-ray and CT scan, MRI can directly demonstrate the disc, spinal cord and the abnormal signal in spinal cord, which is the standard examination for CSM currently [8–10]. Typically, static MRI can only show the neutral and static condition of the spinal cord, which is not adequate for the description of CSM because the cervical motion also plays an important role in the development of CSM [1, 8–13]. Dynamic MRI (dMRI) is firstly described in 1980s, which is a modification of static MRI [14]. Dynamic MRI could show the flexional and extensional position of cervical spine, which is more similar with the natural condition of cervical spine. Some changes of spinal cord would be missed in the static MRI and can be demonstrable in dMRI [13]. Extension MRI helps to identify significant cervical canal stenosis that is partially or completely absent on neutral and flexion MRI. Flexion MRI permits better visualization of hyperintense intramedullary lesions (HILs) on T2-weighted sequence [15]. For patients with cervical canal stenosis (CCS), neck extension may increase the severity of CCS due to the changes of nucleus pulposus, annulus and ligamentum flavum hypertrophy. Cervical flexion shows a relative decrease in total CCS, but the increased stenosis at the most stenotic level is more important [15, 16].

Decompressive and reconstructive surgical techniques for the treatment of CSM may be divided into anterior, posterior, and combined surgical approaches. A systematic review comparing these approaches found similar outcomes that suggest the location of the pathoanatomy may guide the surgical decision making. The choice of which approach and surgical segment to use depends on the desired region of decompression and stabilization [5, 17]. However, how to choose appropriate surgical approach and decompression segments is still controversial [18]. Traditionally, the surgical plan for CSM patients are mainly based on the symptoms and radiographic results [10, 19]. There is growing concerns about the significant value of dMRI for the assessment of CSM. Previous study suggest that preoperative extension MRI can be of great value to determine decompression levels for CSM more accurately [8, 10, 11, 15, 19]. The priorities of dMRI compared to static MRI may modify the surgical decision making and potentially improve the surgical outcome for CSM patients. In this study, we aim to conduct a randomized clinical trial that will evaluate the clinical effectiveness of dMRI in the treatment of CSM compared to static MRI, which is the standard examination currently for CSM patients. Feasibility and validation research will be carried out in our trial to explore the indication and effectiveness of dMRI in the treatment of CSM patients, especially the surgical decision making.

## Methods

### Study design

**Fig. 1:**
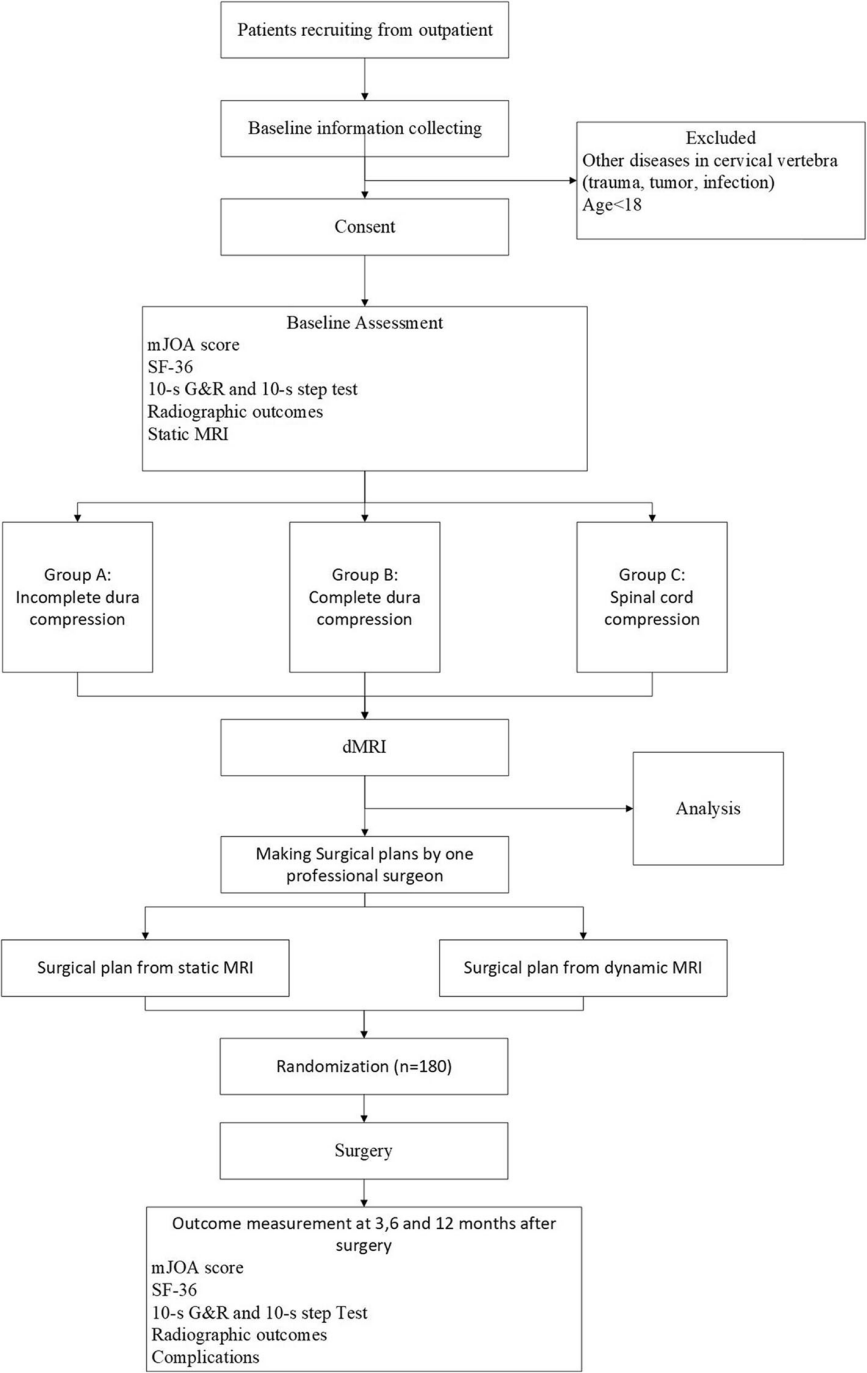
Study design

This study is a prospective, two arm, superiority and open-label randomized controlled trial, including the feasibility research and validation research Fig. 1.

#### Feasibility research

Static MRI will be performed for all patients with CSM who are going to receive surgery. There are three levels of spinal cord compression, incomplete dura compression (Group A, the signal of CSF is still visible), complete dura compression (Group B, no CSF signal and no shape change of spinal cord) and spinal cord compression (Group C, shape change of spinal cord). All participants will be assigned to three groups (A, B, C) based on their static MRI and receive dMRI after consent. The compression degree and T2 high-intensity signal (T2-HIS) on those two MRI will be collected for statistical analysis.

#### Validation research

The professional surgeon will make two surgical plans for each participant according to the static MRI and dMRI, respectively. Then we will randomly choose one via a random number system to perform. Follow-up will be maintained at 3, 6 and 12 months after surgery including modified Japanese Orthopaedic Association score (mJOA), SF-36 score, 10-s G&R and 10-s step test, frontal and lateral radiography of cervical spine and complications.

Finally, we will statistically analyze the difference between static MRI and dynamic MRI to explore the indication of dMRI for CSM patients in the feasibility research. The validation research aims to identify the clinical effectiveness of dMRI in the treatment of CSM patients, especially the surgical decision making.

### Recruitment and informed consent

This single-center study will be conducted in Peking University Third Hospital. Patients with CSM who need surgical treatment will be recruited from outpatient of Peking University Third Hospital. After confirming the eligibility of the patients, the researcher will discuss the study goals, procedures, activities and possible alternatives for approximately 20 min, and answer all questions. After enrolment, participants will be coded as a unique number and general information will be collected.

### Eligibility

The inclusion and exclusion criteria are as follows.

The inclusion criteria:

- ➢ Diagnosis of cervical spondylotic myelopathy (CSM)
- ➢ No surgical contraindications
- ➢ Willing to receive surgery
- ➢ Willing to take part in our study

The exclusion criteria:

- ➢ Other diseases in cervical vertebra (trauma, tumor, infection)
- ➢ Age < 18

### Randomization

Patients will be recruited from outpatient and eligible participants will be enrolled in the study after informed consent. The treating surgeon will also confirm the eligibility before making two surgical plans based on the static MRI and dMRI, respectively. A researcher will randomly choose one from the two surgical plans via a random number system. Randomization of experiment group (dMRI) and control group (static MRI) will be on a 1:1 basis.

### Blinding

As the surgical plans should be informed to patients clearly, the patients cannot be blind to their surgery. However, all patients won’t know which MRI the surgical plan is from. In addition, the treating surgeon will also not be blind to the treatment but will not take part in the preoperative and postoperative research assessment. The clinical outcome data will be collected by independent research assistants who are blind to the surgical decision making.

### Interventions

The surgical indication of Patients with CSM is mainly based on the symptoms, physical and radiographic examination. All participants will receive routine examination at admission, including blood routine and evaluation of general condition. Imaging examination as X-ray, CT and MRI will be accomplished before making surgical plan. All patients will receive imaging examination immediately after surgery and follow-up will be continued at 3, 6 and 12 months after surgery. This trial will involve two types of MRI: static MRI and dynamic MRI. These two types of MRI will be performed in the supine position.

#### Static MRI

Static MRI will just take the neutral position of cervical spine. Static MRI is the routine examination for CSM patients and could demonstrate the disc, vertebrae, cerebral spinal fluid (CSF), spinal cord and the abnormal signal in spinal cord. In this study, the degree of spinal cord compression on static MRI will be assessed according to the three-level classification described above. Also, T2-HIS will be a parameter and collected as exist or non-exist.

#### Dynamic MRI

**Fig. 2:**
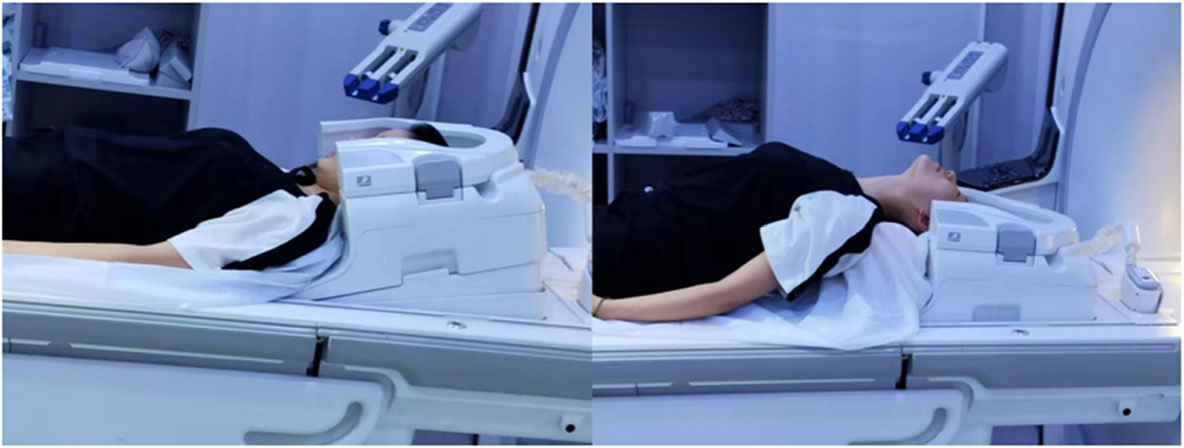
The extension and flexion of cervical spine

Dynamic MRI will show the flexional and extensional position of cervical spine other than neutral position. The angle of extension and flexion is as can be tolerated and it will take longer time than static MRI. The extension and flexion are accomplished by a self-designed posture cushion (Fig. 2). All participants in this study will receive dynamic MRI after informed consent for free. The same evaluation as static MRI will be carried out for dMRI.

#### Surgical intervention

According to the article review by Zhu et al. [20] from our institution, the surgical intervention for CSM was still controversial. Generally, the surgical approaches for CSM can be divided into anterior and posterior approaches. Anterior procedures include discectomy and corpectomy. Posterior procedures include laminoplasty and laminectomy (Table 1). In some cases, combined approach will be necessary.

**Table 1:** surgical options

Surgical approach	Procedures
Anterior	Discectomy and corpectomy
Posterior	Laminoplasty and Laminectomy
Combined	

The level to decompression would be largely determined by patient history, physical examination findings, X-rays, CT, and conventional MRI, and dMRI would always be interpreted with the grain of salt that the compression levels might be increased by dMRI, particularly with neck extension. Additionally, the general condition and expectation of patients will also be taken into consideration before making surgical decision.

### Outcome measures

All participants will be assessed by research assistants who are blinded to the allocation and analysis at different time points shown in Table 2. In addition, the general information including age, gender, weight, symptoms, duration and previous treatment and medication will be confirmed at the admission.

**Table 2:** Time schedule

Measure	Admission	3 months	6 months	12 months
mJOA score	╳	╳	╳	╳
SF-36	╳	╳	╳	╳
G&R and step test	╳	╳	╳	╳
X-ray examination	╳	╳	╳	╳
Static MRI	╳			
Dynamic MRI	╳			

### Primary outcome

#### Cord compression degree

We have discussed the difference of cervical canal stenosis between static MRI and dMRI in the previous study [13]. Dynamic MRI with cervical extension could reveal higher stage of spinal cord compression [9, 11, 15, 21]. All participants in this study will receive static MRI examination after admission and there are three levels of cord compression based on the sagittal and transverse images, including incomplete dura compression, complete dura compression and spinal cord compression. After consent, all participants will receive dynamic MRI examination demonstrating the flexion and extension of cervical spine. The same classification of cord compression will also be applied to dynamic MRI. Meanwhile, the number of levels under moderate to severe compression is also important and we will record this together with cord compression degree.

#### mJOA

The modified Japanese Orthopaedic Association score (mJOA) is a commonly used research tool in the evaluation of cervical myelopathy. It is comprised of 4 domains that evaluate motor function of the upper and lower extremity, sensory function of the upper extremity and bladder function. Previous studies have demonstrated that the mJOA has the validity of evaluation for CSM patients after surgery [22, 23].

The treating clinical team will evaluate the mJOA score for all CSM patients before surgery. The treating clinicians will not be part of the research team. At 3, 6 and 12 months after surgery, mJOA will be assessed via either outpatient or telephone interview during follow-up. The improvement rate of mJOA score at 3, 6, and 12 months compared to baseline will be calculated finally.

### Secondary outcome

#### T2-his

For CSM patients, T2-HIS (T2 high-intensity signal) and decreased signal intensity on T1-weighted MRI are well-known changes in spinal cord lesions. A study showed that the preoperative and postoperative evaluation of CSM patients were better in the patients without T2-HIS compared with patients with T2-HIS. However, T2-HIS grading was not associated with the severity of myelopathy and outcomes, which was reported in the static MRI research [24]. In this study, we will also collect the T2-HIS on the compression segment of static MRI and the extension-flexion position on dMRI, including the length and levels of T2-HIS.

#### SF-36

The SF-36 is a multipurpose, short-form health survey with only 36 questions. It yields an eight-scale profile of scores as well as physical and mental health summary measures [25]. Accordingly, the SF-36 is commonly recommended for the evaluation of quality of life in CSM patients [26]. The treating clinicians will evaluate the SF-36 for all participants after admission. At 3, 6 and 12 months after surgery, SF-36 will also be assessed during follow-up.

#### 10-s G&R and 10-s step test

The mJOA score alone may be insufficient to effectively assess the neurological condition in CSM. The 10-s grip and release (G&R) test and 10-s step test were reported to be useful tools to evaluate the severity of cervical myelopathy quantitatively. These tests were useful for quantitatively evaluating the surgical outcomes in CSM [27, 28].. For the 10-s G&R test, each patient will be asked to G&R with the fingers as rapidly as possible with the forearm kept in pronation and the wrist in mild extension. For the 10-s step test, each patient will be asked to take high steps by bending their knee 90°,making their thighs parallel to the floor. They will be asked to take as many of these steps as they could in place, without holding on to anything for balance for 10-s [24, 29].

#### Radiographic outcomes

The cobb angle between C2 and C7 will be measured before surgery and at 3, 6 and 12 months after surgery during follow-up in the neutral and maximal flexion-extension lateral radiographic view. The fusion and cage subsidence will be recorded as descriptive outcome in the Case Report Form. Cage subsidence in anterior approach is defined as decrease in the total intervertebral height (TIH) between two fused vertebral bodies comparing to the first postoperative radiographs. Usually, decrease in TIH ≥ 3 mm is defined as significant subsidence [30].

#### Complications

All complications and interventions related to the evaluation and treatment for participants will be recorded.

### Adverse event management

Adverse events (AEs) are defined as any untoward medical occurrence in a clinical trial subject and which do not necessarily have a causal relationship with the treatment. In this study, the extra interventions for participants who are willing to receive surgery are dynamic MRI and function assessment, which are non-invasive and safe. The events related to surgery and other diseases which have no correlation with CSM will be counted as the complications and system disease. All participants Experiencing Significant Adverse Events (SAEs) will be followed up as per protocol until the end of the trial.

### Data management

All participants will receive a study number and all data about patients will be recorded via this number. All information about participants including baseline information, radiographic outcomes, surgery and follow-up will be secured in Peking University Third Hospital. Data will be collected at baseline admission and 3, 6 and 12 months after surgery. One professional surgeon will make the surgical plan and finish the surgery in Peking University Third Hospital. The researchers will collect the baseline information and maintain the follow-up after surgery. Data entry, transfer and subsequent maintenance will be performed by a data manager. Access to study data is restricted to the study research team.

### Safety

Participants in this study are going to receive surgical treatment. All extra interventions except surgery in this study are non-invasive but there is the possibility of neurological deterioration with the several minutes for neck extension position, which will be explained to patients when obtaining the consent. The angle of extension and flexion during dMRI is just as the patients can tolerate and there will be a researcher to assist during the examination. Moreover, all expected or unexpected adverse events from this study will be recorded and monitored. Patients suffered from this study will also receive free treatment.

### Sample size calculation

The sample size is calculated with reference to previous study and based on the sample size calculation formula. The average mJOA score improvement rate of surgical treatment according to static MRI in our hospital was 60.8%. we assume that the mJOA score improvement rate12 months after surgery in group dynamic MRI is 85%. We will recruit a sample of 180 participants including three groups stratified according to the spinal cord compression of static MRI. One hundred and eighty is based on a two-sided t-test, a type I error at 0.025 and type II error at 0.1 after taking into consideration a 1:1 allocation rate and a dropout rate of 20%.

### Statistical analysis

Pre-treatment baseline characteristics will be compared between randomized groups to monitor the chance imbalances. Statistical analyses will be performed following the intention-to-treatment (ITT) analysis. The experimental arm (dMRI) will be compared against the control (static MRI) for all primary analysis. We will use chi-squared test for binary outcomes and T-test for continuous outcomes. Spearman’s correlation coefficient will be used to analyze the correlation between the mJOA improvement rate and covariates. A value of *P* < 0.05 will be reported as statistically significant. All analysis will be carried out using SPSS 20.0 software by a researcher blinded to the recruitment and collection.

## Discussion

We have presented the rationale and design of a prospective randomized controlled trial on the therapeutic value of dMRI in CSM patients, especially the surgical decision making. The RCT introduced here includes feasibility and validation research. The feasibility research will compare dynamic MRI with static MRI and explore the indication of dMRI for CSM patients. The validation research will identify the clinical effectiveness of dMRI in the treatment of CSM, especially the surgical decision making.

The long-term follow-up of radiographic outcomes, nerve function recovery and quality of life will provide more evidence about the clinical use of dMRI in the treatment of CSM.

As is noted in some studies, there are many patterns or more cord compression on dMRI of CSM patients which are usually invisible on static MRI and it is significant for the diagnosis and prognostic evaluation for CSM patients [10, 13, 15, 31]. For instance, a prospective study of 50 patients showed that cervical cord available space (CCAS) was greater in the neutral position than in either extension or flexion. The T2-HIS was more evident in flexion position (20/50) than in neutral position (13/50) and in extension (7/50) [10]. Another study demonstrated that higher stages of spinal cord compression were found in the extension position compared with the stage in the neutral and flexion positions. Better visualization of T2-HIS was also found on flexion position [15]. Typically, the T2-HIS and degree of compression on MRI images may reveal the severity of damage of spinal cord [32]. A study shows that the preoperative and postoperative evaluation of CSM patients were better in the patients with no T2-HIS compared with patients with T2-HIS [24]. The specific patterns on the extension and flexion position is a possible reason why there are some patients with negative image findings but still suffer from myelopathy symptoms.

Up to now, limited studies focused on the surgical decision made by dMRI. Two studies of dMRI just mentioned it may be helpful for CSM patients to determine the surgical management by dMRI [8, 15]. Two studies [11, 19] used dMRI in preoperative planning to determine potential levels for decompression. In 1 study [11], upon reviewing the dMRI results, the number of potential levels needing decompression was significant increased by 3 of 4 reviewers with an inter-rater correlation coefficient of 0.81 by dMRI higher than 0.67 by normal MRI. In the other study [19], they had selected the levels requiring decompression with a preoperative dynamic MRI. There was no significant difference of function recovery rate between selective laminoplasty by dMRI and nonselective laminoplasty. This study suggested that a selective decompression based on the preoperative dynamic MRI was clinically useful. However, those studies just focus on the selection of decompression levels and there are also some other factors such as the severity of compression, surgical approach and selection of implants, which should also be taken into consideration when a surgical plan for CSM is made.

Dynamic MRI may play a significant role in the treatment of CSM while there is no enough clinical evidence. The RCT introduced here will have a long period of follow-up after surgery (12 months), comprehensive CSM outcomes, calculated sample size and two stages of research to provide more evidence for clinical effectiveness of dMRI in the treatment of CSM. This new design will help us to modify the surgical decision making of CSM and draw individualized therapeutic schedule for CSM patients according to dMRI.

## Data Availability

The datasets used and/or analyzed during the current study available from the corresponding author on reasonable request.

## Abbreviations

AEs: Adverse events
CCAS: Cervical cord available space
CCS: Cervical spinal canal stenosis
CSF: Cerebral spinal fluid
CSM: Cervical spondylotic myelopathy
CT: Computer tomography
dMRI: Dynamic magnetic resonance image
G&R: Grip and release
HILs: Hyperintense intramedullary lesions
ITT: Intention-to-treatment
mJOA: Modified Japanese Orthopaedic Association
MRI: Magnetic resonance image
RCT: Randomized clinical trial
SAEs: Significant adverse events
SF-36.: Short-Form 36
T2-HIS: T2 high-intensity signal
TIH: Total intervertebral height
